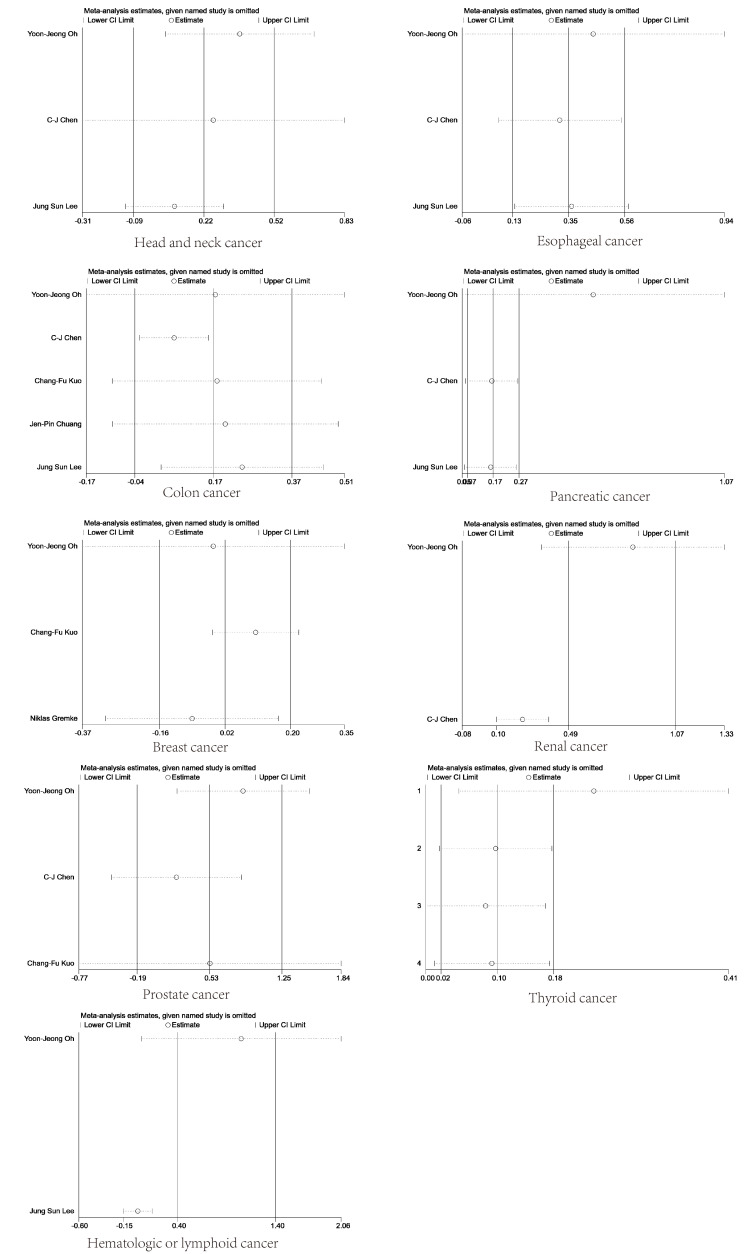# Association between gout and cancers: A systematic review and meta-analysis

**DOI:** 10.1097/MD.0000000000040234

**Published:** 2024-10-25

**Authors:** Lin Tian, Youjiao Wang, Ying Zhang, Lv Tian, Huijing Wang

**Affiliations:** a Department of Thyroid and Breast Surgery, The First Peoples Hospital of Zunyi/Third Affiliated Hospital of Zunyi, Zunyi, Guizhou, China; b Department of Pediatrics, The First Peoples Hospital of Zunyi/Third Affiliated Hospital of Zunyi, Zunyi, Guizhou, China; c Department of Oncology, The First Peoples Hospital of Zunyi/Third Affiliated Hospital of Zunyi, Zunyi, Guizhou, China; d School of Nursing, Jilin University, Changchun, China.

**Keywords:** cancer, gout, meta-analyses, systematic review

## Abstract

**Background:**

This study aimed to investigate the association between gout and cancer risk.

**Methods:**

This study was registered with the Prospective Registry for International Systematic Reviews (ID: CRD42023465587). We searched PubMed, Embase, Scopus, Cochrane, and Web of Science databases for studies related to gout and cancer risk, with a timeframe from the date the database was created to September 2023. We assessed the methodological quality of the included studies using the Newcastle-Ottawa scale and assessed heterogeneity between studies using the *I*^2^ statistic. Depending on the heterogeneity, we calculated pooled hazard ratios (HRs) and corresponding 95% confidence intervals (CIs) using fixed-effects or random-effects models. In addition, we performed sensitivity analyses and publication bias tests.

**Results:**

In this study, we conducted a meta-analysis of 6 studies encompassing a total of 1279,804 participants. Our analysis revealed that individuals with gout are at a heightened risk of developing cancer in general (HR = 1.18, 95% CI = 1.04–1.34, *P* < .001). Moreover, specific types of cancer displayed a significant correlation with gout, including gastric cancer (HR = 1.31, 95% CI = 1.07–1.62, *P* = .012), liver cancer (HR = 1.24, 95% CI = 1.01–1.52, *P* < .001), lung cancer (HR = 1.26, 95% CI = 1.03–1.53, *P* = .001), and bladder cancer (HR = 1.57, 95% CI = 1.02–2.41, *P* < .001). Furthermore, gout exhibited a marginally increased risk for other cancer types, such as head and neck cancer and esophageal cancer, although these associations did not attain statistical significance.

**Conclusion:**

Our study suggests that gout is a risk factor for cancer, especially for stomach, liver, lung, and bladder cancers. Patients with gout have an increased risk of developing overall cancers, lung cancer, liver cancer, stomach cancer, and bladder cancer. However, more high-quality epidemiologic studies are needed to explore the association between gout and individual cancers more accurately.

## 1. Introduction

Gout is a metabolic, immune disease characterized by disturbances in purine and uric acid metabolism. In adults, gout is one of the most common forms of inflammatory arthritis, affecting approximately 9.2 million adults in the United States (3.9% prevalence)^[1]^ and 41.2 million adults worldwide.^[2]^ Gout is caused by the supersaturation of uric acid, which crystallizes within the joints, leading to peripheral joint synovitis and severe pain.^[3,4]^ As the number of acute attacks of gout increases, patients progress to joint damage, deformities, chronic pain, subcutaneous uric acid crystal deposits, and kidney damage. There is a clear association between gout and the metabolic syndrome.^[5,6]^ The prevalence of components of the metabolic syndrome that are associated with gout or hyperuricemia include hypertension,^[6,7]^ insulin resistance/diabetes mellitus,^[7,8]^ obesity,^[6,8]^ and dyslipidemia.^[6]^ The high prevalence of the metabolic syndrome and its components likely contributes to the higher risk of cardiovascular disease,^[7,8]^ emergencies,^[8,9]^ and death in gout patients.^[10]^ The development of gout is often based on long-term metabolic disturbances. There is increasing interest in the relevance of metabolism and immunity in the pathogenesis of gout, and it has been hypothesized that the metabolic syndrome is associated with carcinogenesis.^[5,11]^ In recent years, widespread interest has arisen in the association between gout, hyperuricemia, and cancer. High cellular metabolism may lead to hyperuricemia and tumorigenesis, implying a potential link between disturbances in purine metabolism and cancer.^[12]^ A large body of literature has shown an association between high serum uric acid levels and an independent increase in the risk of cancer.^[13–15]^ In addition, the massive lysis of malignant cells in certain patients with tumor lysis syndrome leads to hyperuricemia, which in turn predisposes to renal failure, coronary heart disease, and gout.^[16,17]^ Interestingly, because of its systemic antioxidant properties, uric acid has been hypothesized to have a protective role in carcinogenesis.^[18]^ However, the association between gout and tumor risk remains controversial. Recent epidemiologic studies have presented conflicting results on the association between gout and cancer risk.^[12,19–23]^ Given these conflicting reports, we conducted a meta-analysis to elucidate cancer risk in patients with gout.

## 2. Methods

### 2.1. Registration information

This study was conducted with the requirements of Preferred Reporting Items for Systematic Review and Meta-Analyses guideline.^[24]^ And it was registered on the International Prospective Register of Systematic Reviews (ID: CRD42023465587).

### 2.2. Search strategy

We conducted a comprehensive search for original studies with gout-cancer correlations from the inception of databases to September 2023 in PubMed, Embase, Scopus, Cochrane Library, and Web of Science. We used the following search terms: ((((cancer*) OR (neoplasm*)) OR (tumor*)) OR (“Neoplasms”[Mesh])) AND (((gout) OR (gouty)) OR (“Gout”[Mesh])). The terms were adjusted according to each database’s syntax and indexing system.

### 2.3. Inclusion and exclusion criteria

Included studies must meet the following criteria:

(1) The study design should be a cohort study or a case-control study.
(2) Studies on the association between gout and cancer risk.
(3) The end-of-period indicator was cancer incidence.
(4) The study reported a hazard ratio (HR) with a corresponding 95% confidence interval (CI) or provided sufficient data to calculate the effect size between gout and cancer.
(5) Published in English.

Studies with the following characteristics were excluded:

(1) The type of article was not reported or could not be determined.
(2) The type of literature was in vivo studies, in vitro studies, or case reports, notes, comments, reviews, and editorials.
(3) Effect sizes could not be extracted or recalculated.
(4) Duplicate studies were also excluded.

### 2.4. Data collection

To ensure the accuracy and objectivity of the data, 2 evaluators (Lv Tian and Lin Tian) independently screened the literature, extracted information, and evaluated the quality of the studies. These processes required cross-checking, and if there was disagreement between the 2 authors, a third person (Youjiao Wang) was consulted to assist in the judgment. Articles were first screened by reading the title, and after eliminating irrelevant literature, abstracts and full texts were further read to determine final inclusion. A predefined data collection form was used to gather information. Data extracted from the studies included first author’s name, year of publication, study design, country, sample size, age of subjects, duration of follow-up, quality assessment scores, type of tumor studied, effect estimates with corresponding 95% CIs, and variables adjusted for in the statistical analysis.

### 2.5. Quality assessment

The Newcastle-Ottawa Quality Assessment Scale is a quality assessment tool for non-randomized studies that evaluates the quality of each study.^[25]^ It consists of 8 items divided into three dimensions, including group selection, group comparability, and exposure/outcome. The checklist has a maximum score of 9, and studies with scores between 7 and 9 are considered high-quality.^[25]^

### 2.6. Statistical analysis

The STATA statistical software (version 15.0; StataCorp, College Station, TX) was used for data analysis. Pooling HRs and 95% CIs assessed the association between gout and cancer risk. *Q* statistic and *I*^2^ statistic were used to assess the degree of heterogeneity of the studies^[26]^ quantitatively. Values of *P* < .10 and *I*^2^ > 50% were considered to represent statistically significant heterogeneity, and the results were then pooled using a random-effects model. Otherwise, forest plots were drawn using a fixed-effects model.^[27]^ Sensitivity analyses were performed by varying the random/fixed-effects model.^[28,29]^ In addition, publication bias could be assessed qualitatively by funnel plot and quantitatively by Begg and Egger tests. *P* values <.05 were considered statistically significant.^[30,31]^

## 3. Results

### 3.1. Study selection and study characteristics

**Figure 1.**
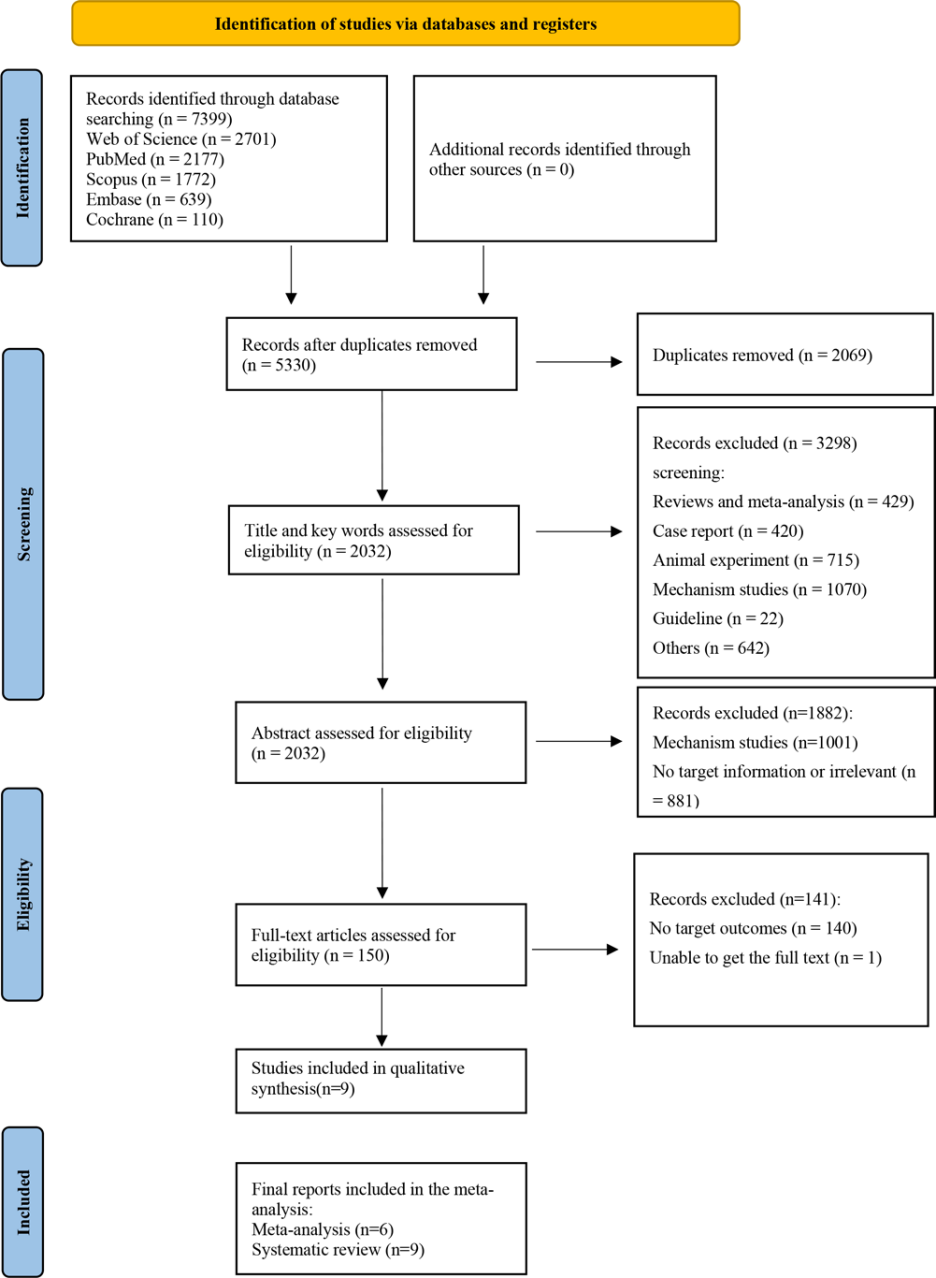
PRISMA flowchart. PRISMA = Preferred Reporting Items for Systematic Review and Meta-Analyses.

A total of 7399 relevant articles were identified through a preliminary search in all search databases based on the search terms provided in Section 2. No additional studies were identified from other sources. A total of 2069 duplicate articles were removed, and 5180 articles were excluded by screening titles or abstracts. In the second stage of full-text screening, we further excluded 141 studies. Ultimately, 9 studies^[12,19–23,32–34]^ were included in this systematic review, of which 6 full-text articles^[12,19–23]^ were available for subsequent data extraction and synthesis. A summary of the inclusion process is shown in Figure 1.

In this meta-analysis, we identified 6 cohort studies^[12,19–23]^ published between 2011 and 2023 with 1279,804 participants. The included studies were all of moderate or high quality. Three of these studies were conducted in Taiwan and China, 2 in South Korea, and 1 in Germany. The population size of the included studies ranged from 12,528 to 694,361. The included studies involved a wide age range of participants, but all were older than 18 years. The articles we included for meta-analysis involved different study populations. Four of the articles examined all types of cancer, while the other 2 focused only on specific types of cancer. There were differences in the distribution of males and females across age groups, with males significantly outnumbering females overall, showing a clear gender difference. The different studies made adjustments for various factors in their analyses. The adjustments made in the studies included various factors such as medical conditions (hypertension, diabetes, dyslipidemia, renal failure, etc), demographic variables (age, sex, and urbanization), lifestyle factors (smoking, drinking, residence, and income), and other comorbidities and clinical measures. Table 1 summarizes the detailed characteristics of the included studies.

**Table 1:** Characteristics of studies included in this study.

Author, year published	Systematic review or meta-analysis	Country	Study design		Age (mean ± SD, Years)	Gender (n, male/female)	Sample size
Yoon-Jeong Oh, 2022^[19]^	Meta-analysis	Korean	Cohort		≥20	283,150 (male), 76,710 (female)	359,860
Niklas Gremke, 2023^[22]^	Meta-analysis	German	Cohort		≥18	67,598 (female)	67,598
Chang-Fu Kuo, 2011^[12]^	Meta-analysis	China, Taiwan	Cohort		42.3 ± 16.3	355,278 (male), 339,083 (female)	694,361
Jen-Pin Chuang, 2019^[21]^	Meta-analysis	China, Taiwan	Cohort		≥19	89,099 (male), 23,210 (female)	112,039
C-J Chen, 2013^[20]^	Meta-analysis	China, Taiwan	Cohort		≥20	33,418 (male)	33,418
Jung Sun Lee, 2020^[23]^	Meta-analysis	Korean	Cohort		41–55	8796 (male), 3732 (female)	12,528
Mats Dehlin, 2022^[32]^	Systematic review	Western Sweden	Cohort		≥18	80,840 (male), 40,161 (female)	121,001
Paolo Boffetta, 2008^[33]^	Systematic review	Sweden	Cohort		≥20	10,500 (male), 6357 (female)	16,857
So Young Kim, 2022^[34]^	Systematic review	Korean	Case-control study		≥40	6170 (male), 23,050 (female)	29,220
Author, year published	Follow-up time (year)	Quailty		Comparisons	Cancer	HR, OR, SIR, IIR (CI)	Adjustment factors
Yoon-Jeong Oh, 2022^[19]^	7	9		Without gout	Multiple cancers	HR: 1.053 (1.031–1.077)	Adjusted*
Niklas Gremke, 2023^[22]^	10	8		Non-gout	Breast cancer	HR: 1.17 (1.05–1.31)	NM
Chang-Fu Kuo, 2011^[12]^	6.6–9.0	9		Non-gout	Multiple cancers	HR: 1.15 (1.10–1.21)	Adjusted†
Jen-Pin Chuang, 2019^[21]^	13	9		Without gout	Colon cancer	HR: 1.03 (0.93–1.14)	Adjusted‡
C-J Chen, 2013^[20]^	8	8		Without gout	Multiple cancers	HR: 2.00 (1.68–2.37)	Adjusted§
Jung Sun Lee, 2020^[23]^	10.1	9		Non-gout	Multiple cancers	HR: 1.224 (1.073–1.398)	Adjusted∥
Mats Dehlin, 2022^[32]^	2–11	NA		Without gout	Multiple cancers	HR: 0.90 (0.84–0.97)	Adjusted¶
Paolo Boffetta, 2008^[33]^	≥1	NA		The National Population of Sweden	Multiple cancers	IIR: 1.25 (1.18, 1.31)	NM
So Young Kim, 2022^[34]^	NM	NA		Thyroid cancer versus non-thyroid cancer	Thyroid cancer	OR: 1.24 (0.99–1.54)	Adjusted#

CI = confidence interval, HR = hazard ratio, IIR = incidence rate ratio, NA = not applicable, NM = not mentioned, OR = odds ratio, SIR = standardized incidence ratio.

* Adjusted for concomitant hypertension, diabetes, dyslipidemia, renal failure, ischemic heart disorder, and cerebrovascular disorder.

† Adjustment for age and sex.

‡ Adjusted model, adjusted for age, sex, urbanization, hypertension, diabetes, hyperlipidemia, CCI, and PS. CCI, Charlson comorbidity index; PS, propensity score.

§ Adjusted for age.

∥ Adjusted for residence, income, smoking, drinking, and comorbidities.

¶ Adjusted for sex and age at baseline, marital status, income, education, born outside of Sweden, alcohol related disorders, hypertension, ischemic heart disease, heart failure, cerebrovascular disease, diabetes mellitus, dyslipidemia, obesity, chronic kidney disease, dementia, lung diseases, and any neoplasm.

# Adjusted for total cholesterol, systolic blood pressure, diastolic blood pressure, fasting blood glucose, obesity, smoking, alcohol consumption, and Charlson comorbidity index scores.

### 3.2. Overall meta-analysis

**Figure 2.**
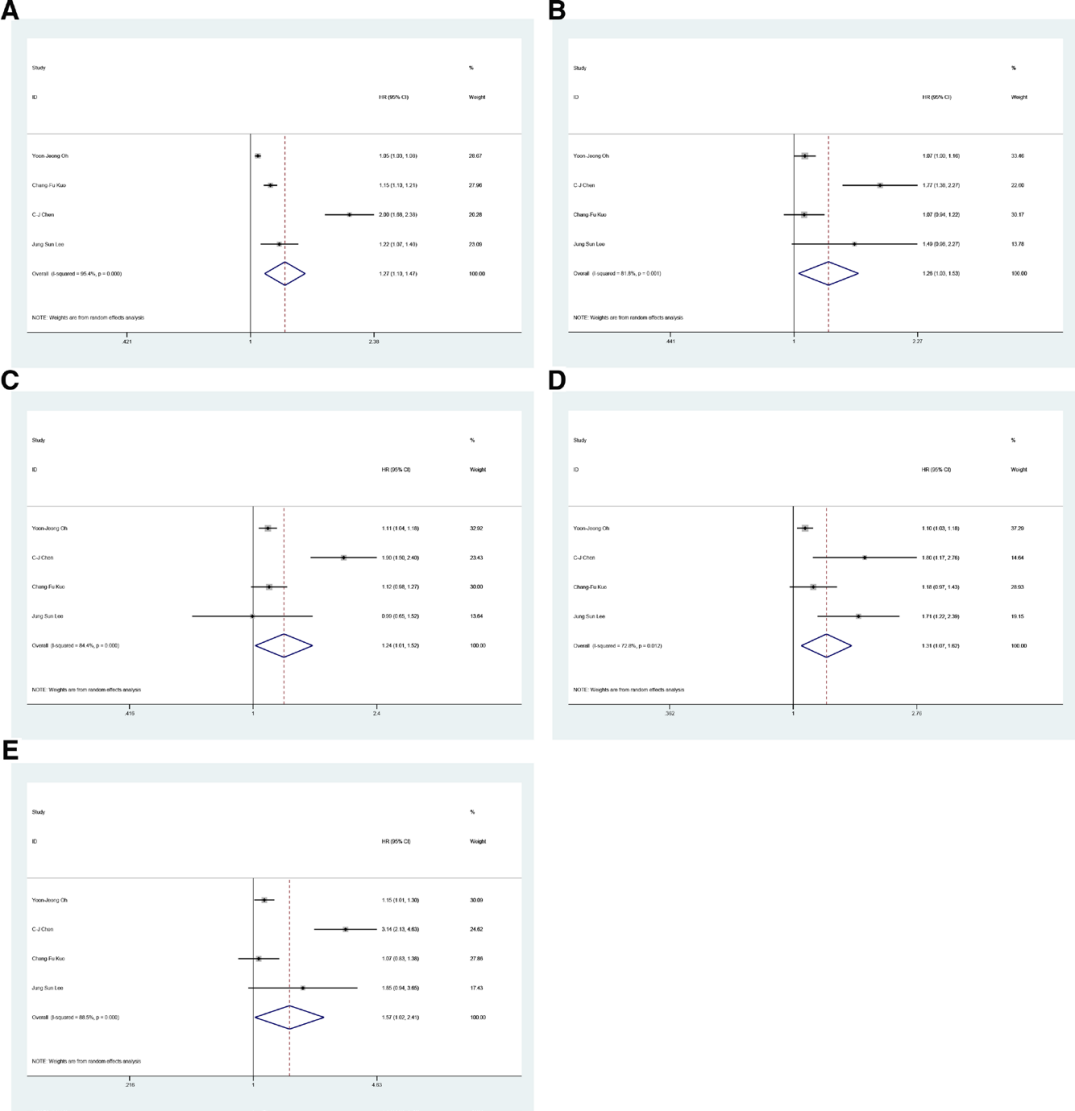
Forrest plot: association between gout and cancer.

Kuo et al^[12]^ reported that the overall incidence of cancer was significantly higher in patients with gout than in controls. Chen et al^[20]^ reported that a study conducted in Taiwan reported that patients with gout were more likely to develop prostate, bladder, colon, kidney, liver, stomach, and lung cancers. Oh et al^[19]^ reported that gout was strongly associated with a higher risk of cancer, especially esophageal, gastric, colon, liver, pancreatic, lung, ovarian, kidney, and bladder cancers. Chuang et al^[21]^ reported that gout does not increase the risk of colon cancer. Gremke et al^[22]^ reported a significant association between gout and development of breast cancer in all populations. Lee et al^[23]^ reported that gout increases the risk of cancer. Six articles^[12,19–23]^ on the association between gout and cancer were included in the meta-analysis. Forest plots show the pooled HRs under the fixed-effects model or the random-effects model. As shown in Figure 2 and Table 2, the pooled results of 4 studies indicated (Fig. 2A) that gout was associated with an increased risk of overall cancers (HR = 1.18, 95% CI = 1.04–1.34, *P* = 0). Among some specific cancer types, the pooled results of 4 studies showed (Fig. 2B) that gout patients had an increased risk of lung cancer (HR = 1.26, 95%CI = 1.03–1.53, *P* = .001), the pooled results of 4 studies showed (Fig. 2C) that gout patients had an increased risk of liver cancer (HR = 1.24, 95% CI = 1.01–1.52, *P* = 0). The pooled results of 4 studies (Fig. 2D) showed that gout patients had an increased risk of gastric cancer (HR = 1.31, 95% CI = 1.07–1.62, *P* = .012). The pooled results of 4 studies (Fig. 2E) showed that gout patients had an increased risk of bladder cancer (HR = 1.57, 95% CI = 1.02–2.41, *P* = 0). The results of all the above studies were statistically significant. Table 2 indicates that gout may have a slight or potential association with an increased risk of head and neck cancer (HR = 1.24, 95% CI = 0.92–1.69, *P* = .086), esophageal cancer (HR = 1.42, 95% CI = 1.14–1.76, *P* = .625), pancreatic cancer (HR = 1.19, 95% CI = 1.08–1.32, *P* = .305), colon cancer (HR = 1.18, 95% CI = 0.96–1.45, *P* = 0), breast cancer (HR = 1.024, 95% CI = 1.96–1.45, *P* = 0), kidney cancer (HR = 1.63, 95% CI = 0.92–2.90, *P* = .023), prostate cancer (HR = 1.7, 95% CI = 0.83–3.50, *P* = 0), thyroid cancer (HR = 1.09, 95% CI = 1.00–1.18, *P* = .484), and hematologic or lymphoid tissue malignancies (HR = 1.49, 95% CI = 0.55–4.04, *P* = .045). However, it is essential to note that these increases did not reach statistical significance.

**Table 2:** Summary of meta-analysis results.

Type of cancer	HR (95% CI)	*I*^2^ (%)	*P* value for heterogeneity analysis
Overall cancers	1.27 (1.10–1.47)	95.4	<.001
Head and neck cancer	1.24 (0.92–1.69)	59.3	.086
Esophageal cancer	1.42 (1.14–1.76)	<0.001	.625
Stomach cancer	1.31 (1.07–1.62)	72.8	.012
Colon cancer	1.18 (0.96–1.45)	91.7	<.001
Liver cancer	1.24 (1.01–1.52)	84.4	<.001
Pancreatic cancer	1.19 (1.08–1.32)	15.8	.305
Lung cancer	1.26 (1.03–1.53)	81.8	.001
Breast cancer	1.024 (0.856–1.225)	72.9	.025
Renal cancer	1.63 (0.92–2.90)	80.6	.023
Prostate cancer	1.7 (0.83–3.50)	98.6	<.001
Bladder cancer	1.57 (1.02–2.41)	88.5	<.001
Thyroid cancer	1.09 (1.00–1.18)	<0.001	.484
Hematologic or lymphoid	1.49 (0.55–4.04)	75.1	.045

CI = confidence interval, HR = hazard ratio.

### 3.3. Sensitivity analyses and bias diagnostics

**Figure 3.**
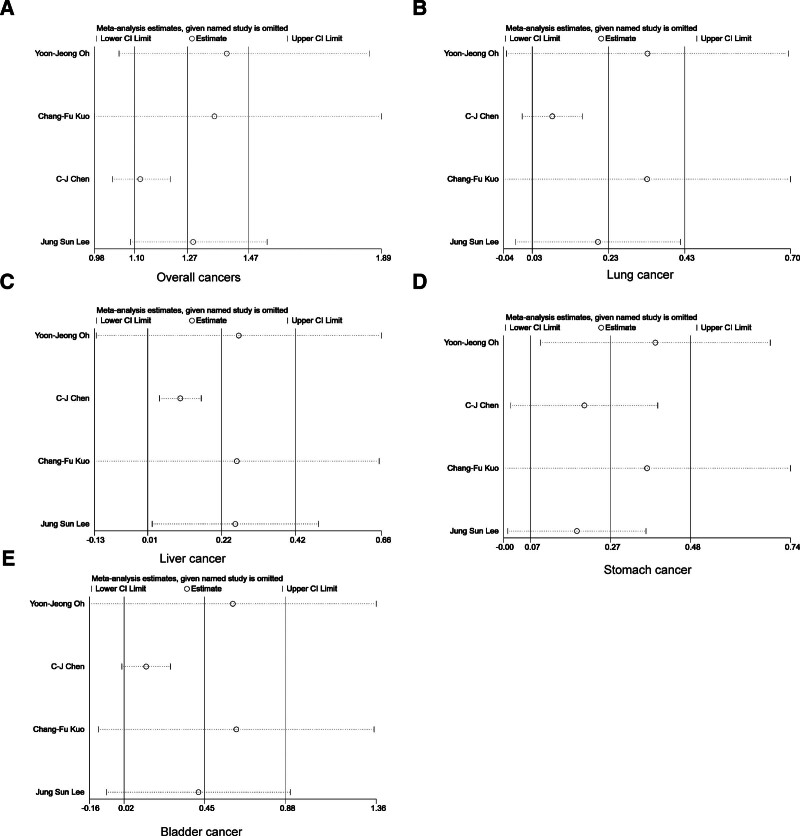
Sensitivity analysis for overall cancers, lung cancer, liver cancer, stomach cancer, and bladder cancer.

We performed sensitivity analyses by varying the random/fixed-effects model, and the pooled results for overall cancers, lung cancer, liver cancer, stomach cancer, and bladder cancer showed that the meta-analysis was statistically stable, as shown in Figure 3A–E. Sensitivity analyses for other specific cancers are shown in Supplementary Material 1, Supplemental Digital Content, http://links.lww.com/MD/N799.

### 3.4. Bias diagnostics

**Figure 4.**
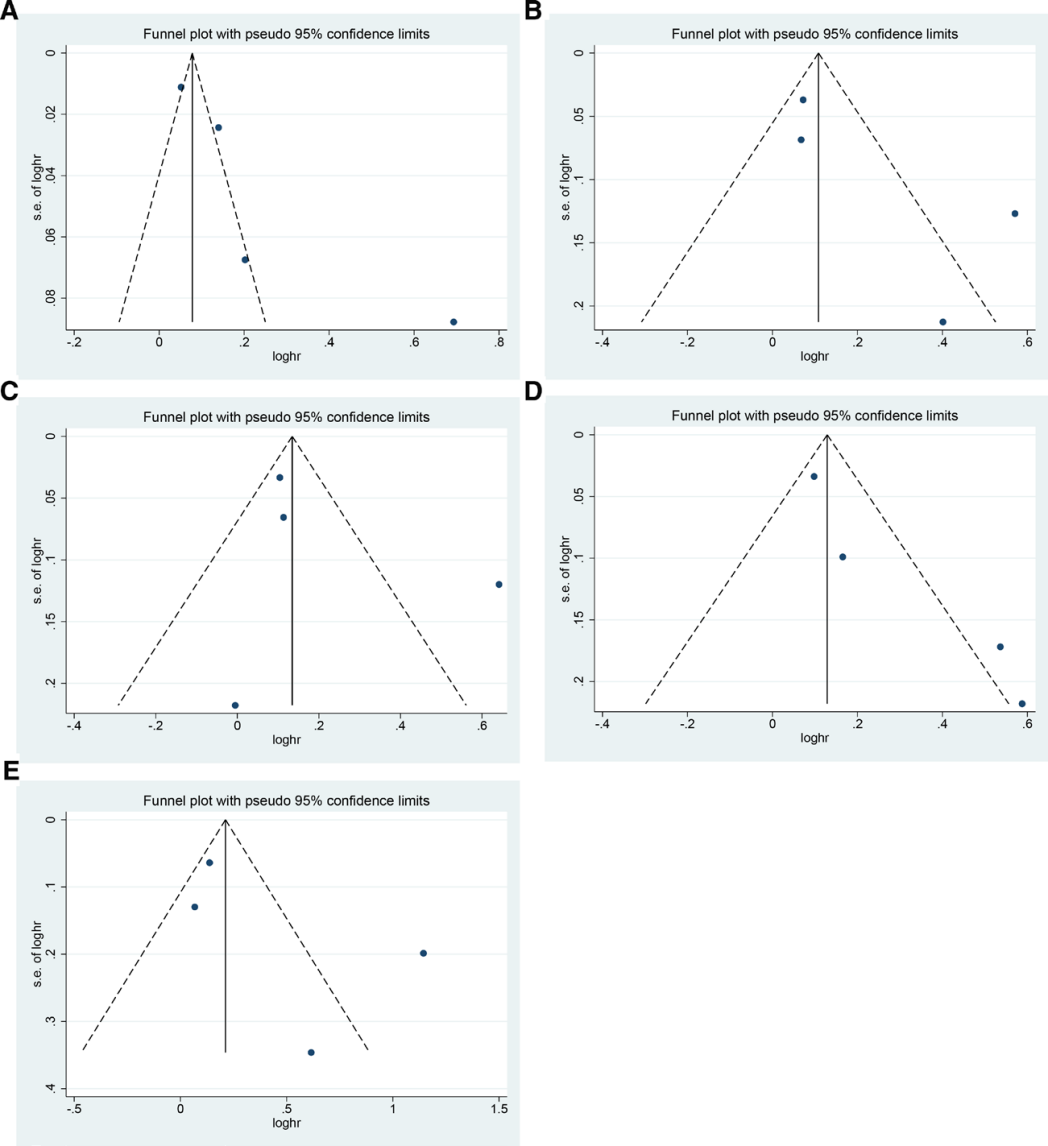
Funnel plot.

No publication bias was found in the association between gout and overall cancers.

(Begg test: *Z* = 0.73, *P* = .462; Egger test: *Z* = 0.99, *P* = .395. The symmetric funnel plot is shown in Fig. 4A). No publication bias was found in the association analysis between gout and lung cancer (Begg test: *Z* = 1.02, *P* = .308; Egger test: *Z* = 1.85, *P* = .206. See Fig. 4B for symmetric funnel plot). No publication bias was found in the association analysis between gout and liver cancer (Begg test: *Z* = 0.34, *P* = .734; Egger test: *Z* = 0.78, *P* = .517. See Fig. 4C for symmetric funnel plot). No publication bias was found in the association analysis between gout and stomach cancer (Begg test: *Z* = 1.02, *P* = .308; Egger test: *Z* = 4.03, *P* = .056. See Fig. 4D for symmetric funnel plot). No publication bias was found in the association analysis between gout and bladder cancer (Begg test: *Z* = 1.02, *P* = .308; Egger test: *Z* = 1.26, *P* = .334. Symmetric funnel plot is shown in Fig. 4E). Other cancer-specific Begg and Egger test values are shown in Table 3.

**Table 3:** Begg and Egger test values.

Type of cancer	*Z*, *P* (Begg test)	*Z*, *P* (Egger test)
Overall cancers	1.02, .308	2.95, .09
Head and neck cancer	1.04, .296	11.94, .053
Esophageal cancer	0, 1	‐0.26, .839
Stomach cancer	1.02, .308	4.03, .056
Colon cancer	‐0.24, 1	0.41, .708
Liver cancer	0.34, .734	0.78, .517
Pancreatic cancer	0, 1	4.33, .144
Lung cancer	1.02, .308	1.85, .206
Breast cancer	1.04, .296	‐14.68, .043
Renal cancer	NA	NA
Prostate cancer	1.04, .296	23.41, .027
Bladder cancer	1.02, .308	1.26, .334
Thyroid cancer	0, 1	4.92, .128
Hematologic or lymphoid	NA	NA

NA = not applicable.

## 4. Discussion

The association between gout and cancer risk has been a controversial topic. Boffetta et al^[33]^ investigated the incidence of cancer in gout patients admitted to a hospital in Sweden and found that gout patients had a higher incidence of oral cavity, pharyngeal, colon, liver, biliary tract, pancreatic, lung, skin, endometrial, and renal cancers than the general population. Kim et al^[34]^ reported that a history of gout was associated with an elevated risk of thyroid cancer in healthy middle-aged people without comorbidities. Previous studies have investigated the association between gout and cancer, but some of them had conflicting findings. The current meta-analysis based on 6 cohort studies showed that we found that gout significantly increased the risk of overall cancer, with consistent findings in specific cancers such as lung cancer, liver cancer, stomach cancer, and bladder cancer. However, our study showed that gout did not alter the risk of developing head and neck cancer, esophageal cancer, colon cancer, pancreatic cancer, breast cancer, kidney cancer, prostate cancer, thyroid cancer, blood or lymphoma. Funnel plot analyses and Begg and Egger test values for overall cancer, gastric cancer, liver cancer, lung cancer, and bladder cancer showed no significant publication bias, which supports the stability of our findings.

The mechanism of association between gout and cancer is not clear, but some studies suggest association. Gout is a disorder of purine metabolism characterized by inflammation and hyperuricemia, which are thought to be associated with carcinogenesis and anticancer, respectively.^[35–37]^ At the same time, some studies have shown that gout is also a chronic inflammatory disease and that a chronic inflammatory state may contribute to the development of tumors.^[38]^ Releasing inflammatory mediators and cytokines may damage DNA, cell proliferation, and tumorigenesis.^[39,40]^ For example, the inflammatory factor IL-1β promotes tumor development by driving chronic unresolved inflammation and endothelial cell activation, affecting angiogenesis and metastasis.^[41]^ Elevated levels of IL-1β have been associated with poor prognosis and aggressiveness in experimental tumor models and a variety of cancers, including melanoma, colon, lung, breast, or head and neck cancer.^[42]^ Meanwhile, uric acid is a key metabolite of gout. On the one hand, some studies have suggested that uric acid is a powerful antioxidant with cancer-fighting properties and a reduced risk of cancer.^[18,43]^ Dehlin et al^[32]^ have reported that gout reduces the risk of cancer mortality. Elevated serum uric acid (normal serum uric acid: 3–6.8 mg/dL) is associated with low cancer risk and cancer mortality, such as prostate cancer, laryngeal squamous cell carcinoma and breast cancer^[44–47]^; on the other hand, some scholars have suggested that gout and hyperuricemia are associated with an increased risk of cancer.^[14,48]^ Certainly, both gout and hyperuricemia are significantly associated with metabolic syndrome, which is thought to be linked to carcinogenesis.^[11]^ Hyperuricemia promotes tumorigenesis by promoting inflammatory stress through reactive oxygen/nitrogen species synthesis and cyclooxygenase activation. After tumor transformation, the free radical scavenging properties of extracellular uric acid protect cancer cells from oxidative stress-induced apoptosis, thereby promoting tumor cell proliferation, migration, and survival.^[15]^ In addition, uric acid induces mesenchymal stromal cell chemotaxis, which is associated with tumor progression and metastasis, and thus may reduce tumor surveillance by promoting the differentiation of immunosuppressive lymphocytes.^[49]^ Furthermore, such factors as unhealthy lifestyle, hypertension, obesity, and dietary habits. These risk factors can increase the risk of cancer in patients with gout.^[50]^ Although we have explored the possible impact of gout on cancer development, the current findings do not clearly explain the specific mechanisms of association between gout and the development of specific types of cancer. Further basic and clinical studies are needed to elucidate the association between gout and cancer.

## 5. Limitations

However, there are some limitations to the meta-analysis we conducted:

1. Six articles were included in our meta-analysis. However, because these articles did not study the same population, some studies examined multiple cancers, and some examined a single type of cancer. Pooled analyses of data on the same cancer included data with sample sizes smaller than 6. For example, only 3 articles addressed data on the correlation between gout and breast cancer, so subgroup analyses by sex, age, country, sample size, and age of subjects were not possible. The insufficient number of original studies also affects our qualitative analysis of incidence bias for specific cancers such as renal, hematologic, or lymphomas, so our results should be interpreted cautiously.
2. The effect of confounders on our findings was not further elucidated.
3. There was heterogeneity among the included studies, so we used a random-effects model to combine HRs.
4. Because all included studies were observational, we could not determine the effect of specific causes on the results. Cancer is a multifactorial disease with complex causative factors, including genetic polymorphisms, environmental carcinogens, family history, and chronic inflammatory diseases.^[51]^ Interactions between gout and other carcinogens may be a pivotal contributor to carcinogenesis, which needs to be elucidated by further studies.

Despite these limitations, our meta-analysis has some significant strengths:

1. The sensitivity analysis showed that the meta-analysis results did not change substantially when we used the “leave-one-out” method, so the results were reliable. Begg and Egger tests showed that there was no publication bias.
2. We included more studies than in previous meta-analyses, which increased statistical power, and we conducted a more comprehensive meta-analysis of different cancer types.
3. We also explored possible common mechanisms of action of gout and cancer, which provided a better theoretical basis for the meta-analysis.

## 6. Conclusion

In summary, our study suggests that gout increases the risk of cancer, particularly stomach, liver, lung, and bladder cancers. However, more well-designed basic and clinical studies are needed to explore these associations in depth. For clinicians, we recommend regular and close monitoring of patients to detect occult malignancies and improve the quality of care for gout patients.

## Author contributions

**Conceptualization:** Lin Tian.

**Data curation:** Youjiao Wang.

**Formal analysis:** Ying Zhang.

**Funding acquisition:** Huijing Wang.

**Investigation:** Lv Tian.

**Project administration:** Lin Tian.

**Resources:** Youjiao Wang.

**Software:** Ying Zhang.

**Supervision:** Lv Tian.

**Validation:** Huijing Wang.

**Writing – original draft:** Lin Tian.

**Writing – review & editing:** Youjiao Wang.

## Supplementary Material

**Figure:**